# Mutual Information between Discrete Variables with Many Categories using Recursive Adaptive Partitioning

**DOI:** 10.1038/srep10981

**Published:** 2015-06-05

**Authors:** Junhee Seok, Yeong Seon Kang

**Affiliations:** 1 School of Electrical Engineering, Korea University, Seoul, South Korea; 2 Department of Business Administration, University of Seoul, Seoul, South Korea

## Abstract

Mutual information, a general measure of the relatedness between two random variables, has been actively used in the analysis of biomedical data. The mutual information between two discrete variables is conventionally calculated by their joint probabilities estimated from the frequency of observed samples in each combination of variable categories. However, this conventional approach is no longer efficient for discrete variables with many categories, which can be easily found in large-scale biomedical data such as diagnosis codes, drug compounds, and genotypes. Here, we propose a method to provide stable estimations for the mutual information between discrete variables with many categories. Simulation studies showed that the proposed method reduced the estimation errors by 45 folds and improved the correlation coefficients with true values by 99 folds, compared with the conventional calculation of mutual information. The proposed method was also demonstrated through a case study for diagnostic data in electronic health records. This method is expected to be useful in the analysis of various biomedical data with discrete variables.

Mutual information is a statistic to measure the relatedness between two variables^1^. It provides a general measure based on the joint probabilities of two variables assuming no underlying relationship such as linearity. Compared with traditional measures such as correlation, mutual information can detect a wider range of relationships. For example, zero correlation coefficient does not necessarily imply that two variables are independent while zero mutual information is mathematically equivalent to independence. Mutual information has a strong theoretical ground in information theory^2^, which has been intensively studied and applied in the various areas of informatics, engineering, communications, and computer science (reviewed by Cover and Thomas^1^). In addition, mutual information is directly interpreted as the amount of shared information between two data sets in the unit of bits. Benefiting from such advantages, mutual information has been actively utilized in the analyses of biomedical data; sequence analysis^3^, gene expression analysis^4^,^5^,^6^, molecular network reconstruction^7^,^8^, biomedical image processing^9^, and the analyses of electronic health records^10^.

Although the calculation of mutual information is straightforward given a joint probability distribution of two variables, in many cases it should be estimated from random samples without knowing the underlying distribution. There have been numerous efforts to estimate mutual information from random samples, especially for continuous variables. A common way is a binning approach using two-dimensional histogram, where the whole sample space is separated into several bins and the joint probabilities are estimated by counting the number of samples in each bin^11^,^12^. To improve the efficiency of binning, partitioning after data transformation to a favored coordinate system has been suggested^13^. As an alternative to the binning approaches, algorithms estimating the joint probability distribution with smoothing functions or kernels have been studied^14^.

Until now, many studies for mutual information have focused on its calculation between continuous variables. While the mutual information between discrete and continuous variables has been studied recently^15^, mutual information between discrete variables hasn’t been studied much. It is mainly because the estimation of the joint probabilities between discrete variables has been considered to be straightforward, just by counting the number of samples in each combination of categories of two variables^15^. In fact, even without sophisticated techniques, the conventional calculation of mutual information works well for discrete variables of a few categories with a relatively large sample size.

With recent developments in data generation, storage, and management, new types of data with many categories have been introduced. For example, electronic health records have diagnosis codes, operations, and prescribed drugs, each of which has thousands of different categories. In order to investigate their relations, we have to examine millions of distinct combinations with a limited sample size. In population genomics, the genotype of a SNP (single nucleotide polymorphism) position can be encoded as a discrete variable with three values (AA, AB, and BB). However, when we consider *n* SNPs together, the number of genotype categories exponentially increases. In an association study between genotypes and phenotypes, such as phenome-wide association studies that consider many phenotypes together^16^, several millions of possible combinations may need to be explored. In genome sequence analyses, while a nucleotide can have only four categories (A, C, G and T), a codon, three consecutive nucleotides as a unit of amino acid translation, can have 64 distinct values. In immunology, the VDJ recombination of B-cell and T-cell receptors is a key mechanism for the variety of immune repertoires. The investigation of recombination patterns through immune repertoire sequencing^17^ requires examining the combinations of ~80 V, ~30 D, and ~6 J segments. Examples of available data sets are listed in Supplementary Table S1. These data types commonly have many categories or discrete values, among which orders and distances are ill-defined.

For such discrete variables with many categories, the conventional calculation of mutual information based on frequencies of all possible combinations is not efficient. It has been known that in the binning approaches^11^,^12^, the calculated mutual information between two continuous variables depends on quantization and sample sizes^18^, and it can be distorted if too many bins are used^15^. Similarly, the calculation of mutual information between discrete variables with many categories can be easily distorted, simply because there are only a few observations for each combination of categories. For the continuous variables, this problem, at least partially, can be solved by techniques based on data transformation and smoothing^13^,^14^. However, for discrete variables with fixed categories without an order, there is no room to apply such techniques. This problem still remains unsolved for discrete variables and needs to be explored more.

In this paper, we propose a method to calculate the mutual information between two discrete variables with many categories by using recursive adaptive partitioning. The method is for discrete variables of which values are not orderable. The conventional approach suffers from the relative sparseness of observed samples because there are too many possible combinations of the categorical values of two discrete variables. By recursively rearranging categories in the order of marginal populations and partitioning the sample spaces, the method separates the whole sample space into much fewer subregions within which the samples are considered to be uniformly distributed. Since all samples in a subregion are considered together regardless of their actual combinations of categorical values, the proposed method virtually reduces the effective number of possible choices, and consequently increases the relative sample density. The proposed method was evaluated by intensive simulation studies, where it showed superior robustness and accuracy compared with the conventional calculation. As a case study, this method was applied to the analysis of electronic health records of intensive care unit patients in MIMIC-II Clinical Database^19^. The results of both simulation and case studies show the usefulness of the proposed method in the analysis of relation between discrete variables with many categories.

## Methods

### Conventional Calculation of Mutual Information

Consider two discrete variable *X* and *Y* with *x*_1_, *x*_2_, …, *x*_*n*_, and *y*_1_, *y*_2_, …, *y*_*m*_ distinct values or categories, respectively. Let *N*(*x*_*i*_,*y*_*j*_) denote the number of samples with *x*_*i*_ and *y*_*j*_ values, and *N*_*T*_ be the total number of samples. The joint probability mass *p*(*x*_*i*_, *y*_*j*_) = Pr(*X *= *x*_*i*_,*Y* = *y*_*j*_) is often empirically estimated by the number of samples with *x*_*i*_ and *y*_*j*_, i.e. *p*(*x*_*i*_, *y*_*j*_) = *N*(*x*_*i*_,*y*_*j*_)/*N*_*T*_. The marginal probability mass *p*(*x*_*i*_) and *p*(*y*_*j*_) can be accordingly calculated. Then, the mutual information between *X* and *Y*, *I*(*X*;*Y*), is calculated by


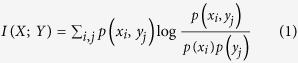


with the naively estimated joint probabilities^1^. The mutual information of *X* and *Y* is 0 if they are independent.

### Recursive Adaptive Partitioning

**Figure 1:**
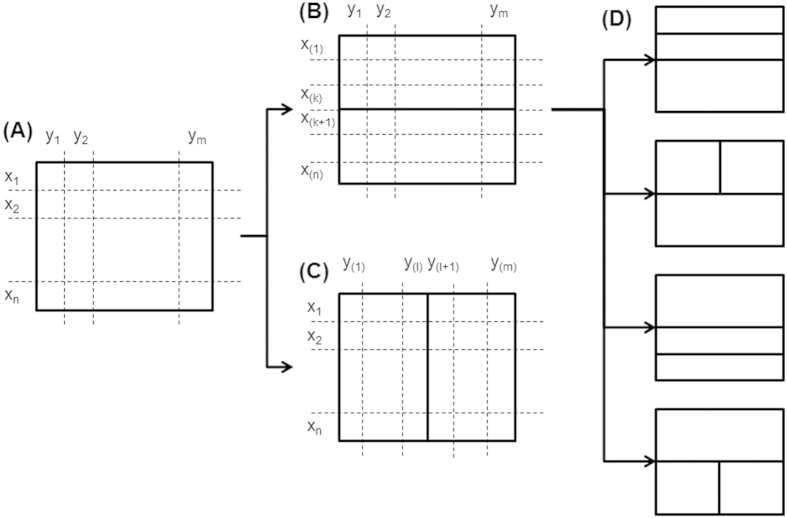
Procedure of recursive adaptive partitioning. (**A**) A given subspace of data with two discrete variables *X* and *Y* are shown. *X* has *n* distinct categories of *x*_1_ to *x*_*n*_, and *Y* has *m* categories of *y*_1_ to *y*_*m*_. (**B**) The first partitioning for *X* is made between *x*_(*k*)_ and *x*_(*k*+1)_. *x*_(*i*)_ represents the *i*-th categorical value of *X* in the order of the marginal populations. (**C**) The first partitioning for *Y* which is made between *y*_(*l*)_ and *y*_(*l*+1)_. (**D**) In the case that the first partitioning is made for *X*, the potential results of the second partitioning are shown.

For the calculation of mutual information between two discrete variables with many categories, we first partition the whole sample space into several subregions where samples are supposed to be uniformly distributed. Categorical values cannot be ordered by themselves. Instead, here we sort them in the order of their marginal populations. Figure 1 illustrates the procedure of the proposed recursive adaptive partitioning. As shown in Fig. 1A, consider a subspace where discrete variable *X* and *Y* have *n* and *m* distinct categories, respectively. This subspace exclusively has *mn* possible combinations of *X* and *Y* categories. This subspace is further partitioned through the following steps.

1. We test the uniformity of the sample distribution in the subspace using a chi-square statistic^20^. The chi-square statistic is calculated by


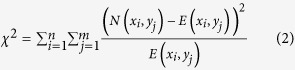


where *E*(*x*_*i*_,*y*_*j*_) denote the expected number of samples with *x*_*i*_ and *y*_*j*_ categories when the samples are uniformly distributed, i.e. *E*(*x*_*i*_,*y*_*j*_) = Σ_*i,j*_*N*(*x*_*i*_,*y*_*j*_)/*nm*. The corresponding *p*-value is calculated under a chi-square distribution with the degree of freedom of *nm*−1. If the *p*-value is larger than a certain threshold, then the sample distribution is considered to be uniform. In this case, we stop at this point and no further partitioning is made. Otherwise, since the samples are not uniformly distributed, the subspace is further partitioned by the next steps.

2. The subspace can be partitioned for either *X* (Fig. 1B) or *Y* (Fig. 1C). The proposed method splits a subspace into two regions (binary partitioning) at the optimal point that minimizes the mean-square-errors when the uniformity is assumed. To find the optimal split point for *X*, we sort *x*_*i*_’s, the categorical values of *X*, in the order of their marginal populations within the subspace. The marginal population of *x*_*i*_ is given as *N*_*M*_(*x*_*i*_) = Σ_*j*_*N*(*x*_*i*_,*y*_*j*_). We present the ordered values as *x*_(1)_, *x*_(2)_, …, *x*_(*n*)_ where *N*_*M*_(*x*_(1)_) ≤ *N*_*M*_(*x*_(2)_) ≤ … ≤ *N*_*M*_(*x*_(*n*)_). Then, the optimal split point *s*_*X*_ for *X* can be found by minimizing *T*_*X*_ given as


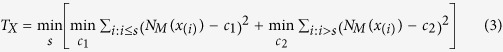


where *c*_1_ and *c*_2_ are naturally given by the averages of the corresponding *N*_*M*_(*x*_*i*_)’s. Similarly, we can calculate the optimal split point *s*_*Y*_ for *Y* and *T*_*Y*_. By comparing *T*_*X*_ and *T*_*Y*_, the given subspace (Fig. 1A) is partitioned for *X* at *s*_*X*_ if *T*_*X*_>*T*_*Y*_ (Fig. 1B). Otherwise, it is partitioned for *Y* at *s*_*Y*_ (Fig. 1C). Since the categories are rearranged at every step of recursion, the pre-arrangement of categorical values before the analysis does not affect the result.

3. Each of the partitioned subspaces is considered as a new subspace. Step 1 and 2 are repeated for these new subspaces. Figure 1D shows the potential partitions in the next round when the subspace of Fig. 1A is partitioned for *X*.

The proposed method assumes that unobserved proper categories can be efficiently found using marginal populations. The method aims to find blocks within which observed samples are uniformly distributed. These blocks can be considered as unobserved proper categories because the relation of two variables can be captured by these blocks instead of the fine combinations of observed categories. However, since it is difficult to directly find such jointly uniform blocks, the proposed method uses the marginal uniformity. While the marginal uniformity does not guarantee the joint uniformity, partitioning by maximizing the marginal uniformity of the separated subregions can help to extract the jointly uniform regions because the joint uniformity guarantees the marginal uniformity. Especially, by recursively applying this maximization and partitioning procedure, the jointly uniform blocks are expected to be finally found in many practical cases.

### The Proposed Calculation of Mutual Information

Through the recursive adaptive partitioning, the whole sample space is partitioned into non-overlapping subregions. Subregion *A* is defined by exclusive combinations of *X* and *Y* categories, i.e.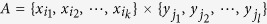. The total probability mass assigned to *A* is given as 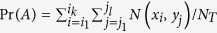 where *N*_*T*_ is the total number of samples. Since the samples are considered to be uniformly distributed in *A*, the joint probability mass of *x*_*i*_ and *y*_*j*_ is given as *p*(*x*_*i*_, *y*_*j*_) = Pr(*A*)/*kl*. Using this joint probability masses, we can calculate the corresponding marginal probabilities. Finally, we can calculate the mutual information as equation (1). Note that if all subregion *A*’s have only one combination of *x*_*i*_ and *y*_*j*_, i.e. *k* = *l* = 1, the proposed calculation is equivalent to the conventional one.

### Related Previous Works

While the proposed method is for discrete variables, similar ideas of adaptive partitioning have been introduced for the estimation of mutual information between continuous variables^21^,^22^. They recursively divide a continuous sample space until the sample distribution uniformity within partitions is confirmed. Then, they estimate the joint probability distribution and calculate mutual information. Such methods often find the partitioning points at certain quantiles, such as median (50% quantile), of the cumulative probability of each variable, which is not possible for discrete variables whose categorical values have no order. Instead, the proposed method finds the partitioning point over the sorted categories in the order of marginal populations. Moreover, the methods for continuous variables require to partition one step further to check the uniformity of the sample distribution because the uniformity in a region cannot be determined without discretization. In contrast, our method checks the uniformity over the combinations of categories in a subregion without further partitioning. Even with some similarity of the basic idea, our method for discrete variables is quite distinct from the previous ones for continuous variables.

For mutual information between continuous variables, as an alternative of adaptive partitioning, the use of regular partitioning with bias correction has been proposed^23^. These methods divide a continuous sample space into equal-size partitions, estimated the joint probability density, and calculate entropies and mutual information by correcting partitioning biases using polynomial approximations. While the idea of the bias correction is developed for continuous variables, it might be possible to apply for discrete variables as an alternative of the adaptive partitioning described in this work.

The adaptive partitioning approach also has been used to improve the sampling strategy of pixel densities in the estimation of the mutual information between two images^24^. Here, an image is spatially adaptively partitioned by a recursive process. By sampling pixel densities from such partitions, the probability distribution of the pixel density of the image can be estimated with the consideration of spatial patterns. However, the mutual information between the pixel density distributions of two images is simply estimated in the conventional way^1^. This method applies the adaptive partitioning for sampling before the calculation of mutual information while the proposed method directly applies for the mutual information calculation.

## Results And Discussion

### Simulation Results

**Figure 2:**
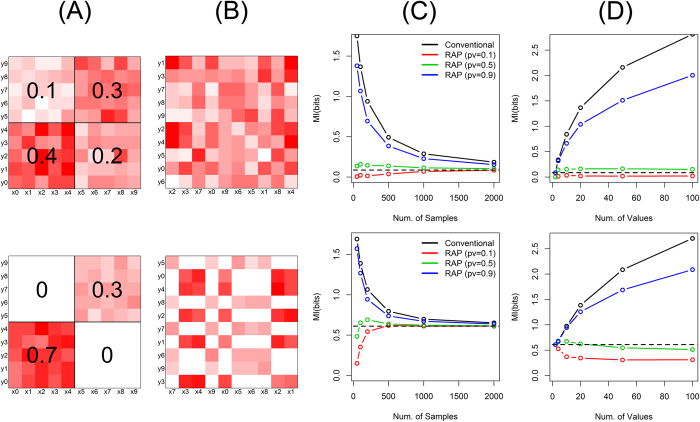
Mutual information of step structure. The upper panels are for simulation setting (1) and the lower panels are for (2). (**A**) Each variable has 10 distinct categories, and their joint probability masses are structured by step functions. (**B**) Observed data with randomly ordered categorical values. (**C**) Mutual information as a function of sample sizes, by the conventional method (black) as well as by the proposed method (RAP: recursive adaptive partitioning) with different *p*-value thresholds (red: 0.1, green: 0.5, blue: 0.9). The true mutual information is shown with a dashed black line. (**D**) Mutual information as the numbers of distinct categories for each variable. 10, 50, 100, 250 and 500 samples were used for 2, 10, 20, 50 and 100 categories, respectively.

The proposed method was evaluated through simulation studies with discrete variables of which values cannot be ordered. Simulation settings are described in Supplementary Methods. Figure 2 shows the simulation results for data structured by step functions (simulation setting (1) and (2) in Supplementary Methods). In this simulation, variable *X* and *Y* are considered to have two super categories for each. Their joint probability masses are shown in Fig. 2A. Instead of the super categories, five categories per each super category are assumed to be observed. The combinations of the observed categories are assumed to be uniformly distributed within the corresponding combination of the super categories. Since the categorical values have no order, we observe 10 × 10 complicated combination patterns between *X* and *Y* instead of 2 × 2 simple pattern (Fig. 2B). This simulation reflects cases that we observe and analyze fine categories while truly informative super categories are unobserved.

The proposed calculation of mutual information with recursive adaptive partitioning provides much closer estimations than the conventional calculation in a wide range of simulation parameters. Figure 2C shows the calculated mutual information for 20 categories for each variable as the numbers of samples. All tested calculations converge to the true value with the increase of sample sizes. While varying with the *p*-value thresholds, our method shows faster convergences than the conventional calculation, and closer estimations to the true value at a given sample size. In the example of the lower panel of Fig. 2C where the true mutual information is 0.61 bits, the estimated mutual information by the proposed method is 0.69 bits (sample size = 200, *p*-value threshold = 0.5), and the error is 0.08 bits. For the same setting, the conventional calculation provides 1.07 bits as its estimation, and the error is 0.56 bits, which is seven times larger than that of the proposed method.

The proposed estimation is robust to the number of distinct categories of variables. Figure 2D shows the change of the estimated mutual information according to the numbers of categories for variable *X* and *Y*. With the increase of the number of categories, conventionally calculated mutual information is dramatically deviated from the true value. In the example of the upper panel of Fig. 2D, the mutual information is conventionally calculated as 2.81 bits for the 100 categories per variable, and the error to the true value (0.09 bits) is 2.72 bits. In contrast, the proposed method provides stable estimations even with very many categories. For the same 100 categories, its estimation is 0.14 bits (*p*-value threshold = 0.5) and the error is 0.05 bits, which is more than a 50-fold deduction from the conventional calculation.

**Figure 3:**
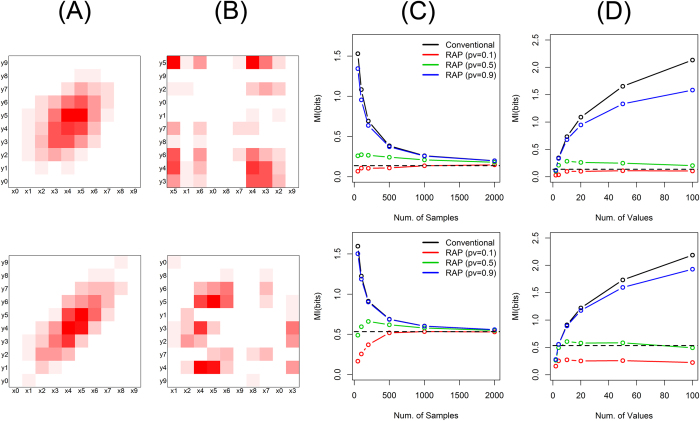
Mutual information of Gaussian structure. The upper panels are for simulation setting (3) and the lower panels are for (4). (**A**) Each variable has 10 distinct categories, and their joint probability masses are structured by a joint Gaussian function. (**B**) Observed data with randomly ordered categorical values. (**C**) Mutual information as the numbers of samples, by the conventional method (black) as well as by the proposed method (RAP: recursive adaptive partitioning) with different *p*-value thresholds (red: 0.1, green: 0.5, blue: 0.9). The true mutual information is shown with a dashed black line. (**D**) Mutual information as the numbers of distinct categories for each variable. 10, 50, 100, 250 and 500 samples were used for 2, 10, 20, 50 and 100 categories, respectively.

**Figure 4:**
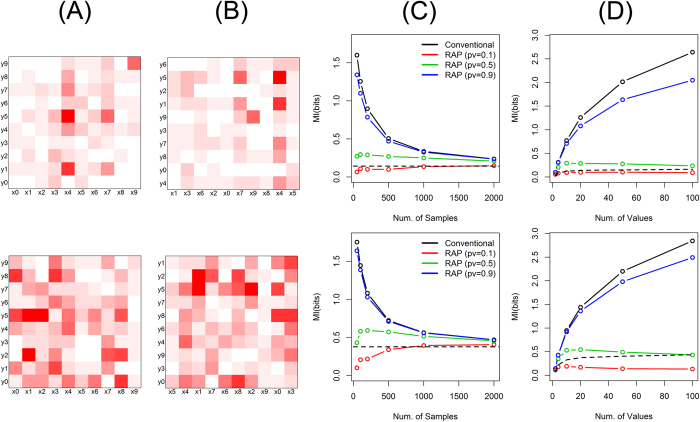
Mutual information of random structure. The upper panels are for simulation setting (5) and the lower panels are for (6). (**A**) Each variable has 10 distinct categories, and their joint probability masses are randomly structured. (**B**) Observed data with randomly ordered categorical values. (**C**) Mutual information as the numbers of samples, by the conventional method (black) as well as by the proposed method (RAP: recursive adaptive partitioning) with different *p*-value thresholds (red: 0.1, green: 0.5, blue: 0.9). The true mutual information is shown with a dashed black line. (**D**) Mutual information as the numbers of distinct categories for each variable. 10, 50, 100, 250 and 500 samples were used for 2, 10, 20, 50 and 100 categories, respectively.

Simulation studies with other data structures also show the similar accuracy and robustness of the proposed method. Figure 3 shows data where the joint histogram of two categorical variables is characterized by a joint Gaussian distribution (simulation setting (3) and (4) in Supplementary Methods). It reflects the case that the category populations of variables have bell shapes marginally and they are correlated with each other. The data structure can be easily recognized with properly arranged categorical values (Fig. 3A). However, when the data is actually observed, the structure is difficult to be perceived because the categories are likely to be randomly arranged without an order (Fig. 3B). Figure 4 shows the randomly structured data, where each joint probability is randomly chosen from exponential distributions (simulation setting (5) and (6) in Supplementary Methods). As a result, the designed data structure (Fig. 4A) has visually no significant difference from the observed data (Fig. 4B). Since the theoretical mutual information is difficult to be calculated in these cases, we empirically estimated the true values with very large sample sizes. For both data sets with Gaussian and random structures, the proposed method provides close estimations to the true values even with small sample sizes (Figs. 3C,4C) as well as robust estimations even with many categories per variable (Figs. 3D,4D).

The systematic evaluation is summarized in Table 1 for the cases with 500 samples and 100 categories per variable. Table 1 also reports the average ratios of the errors to the true values (|MI_true_−MI_estimated_|/MI_true_) as well as the Pearson’s correlation coefficients between the estimated and true mutual information values of the six simulations. The conventional method always over-estimates the mutual information. Data sets with low and high mutual information, such as (1) vs. (2), are hardly distinguishable with the conventional method. In contrast, the proposed method achieves significantly smaller errors and higher correlations with the true values. The performance of the proposed method varies as *p*-value thresholds. Although it is very difficult to guess the optimal p-value threshold or to derive a parameter-free way of estimating it, one can choose a threshold from a wide range of p-values that shows reasonably good performance. Especially, our method with *p*-value threshold 0.5 shows the best performance in the simulations with low errors (0.27 bits on average) as well as high correlations with true values (0.99). Compared with the results of the conventional methods (error: 12.20 bits, correlation: 0.01), the average error was reduced by 45 folds and the correlation coefficient was improved by 99 folds, which are significant improvements.

We also compared our method with random partitioning to evaluate the efficiency of our partitioning scheme. In the random partitioning, one of *X* and *Y* is randomly chosen for split, and the corresponding categories are randomly separated into two groups. Table 1 shows the result of random partitioning with the same number of final subregions with the proposed method with *p*-value threshold 0.5. Random partitioning hardly captures the relation between variables, and provides almost zero mutual information for all cases. This comparison shows that our method efficiently learns the relation of variables through adaptive partitioning and consequently provides accurate estimations.

Additionally, we investigated the noise effects on the mutual information calculation. The noise of a discrete variable can be interpreted as mistakes in categorization. The noise level can be measured as the proportion of these mistakes among the total samples. Since a noise sample can be mis-categorized as any value, noise samples are distributed uniformly over the whole sample space. Therefore, mutual information between two discrete random variables is distorted toward zero by noise. Using the simulation setting (4), we evaluated the noise effect (Supplementary Figure S1). The expected mutual information was estimated from 1 million samples using the conventional method at each noise ratio. The mutual information was estimated from subsampling by the proposed method (p-value = 0.5) and the conventional method. As shown in Supplementary Figure S1, the expected mutual information was distorted toward zero by noise. The estimation by our method followed the expected mutual information well while the conventional method hardly captured the noise effect. The proposed method can sensitively capture the distortion of mutual information by noise.

### A Case Study for the Diagnoses in Critical Care

For a case study, we applied the proposed method to investigate the relation between the primary and secondary diagnoses of intensive care unit (ICU) patients. 4,928 pairs of primary and secondary diagnoses for admissions were collected from 3,854 patients in the publically available subset of MIMIC-II Clinical Database^19^. Diagnoses are represented by the 3-digit ICD9 (International Classification of Diseases 9) codes (www.icd9data.com). Among about a thousand disease codes in the ICD9 codes, the primary and secondary diagnoses in the data have 296 and 252 disease codes, respectively. Since the disease codes are discrete and have no order, the primary and secondary diagnoses can be represented as discrete variables with those numbers of distinct categories.

We estimated the mutual information between the primary and secondary diagnoses. For the proposed method, we used 0.5 as a *p*-value threshold because it showed the reasonably good performance through the simulation studies. The mutual information was estimated as 0.34 bits by the proposed method. In contrast, it was estimated as 1.13 bits by the conventional calculation. Since the true value is unknown, it is difficult to evaluate the accuracy of these estimations. However, on average there are 0.066 (=4,928/296/252) samples per distinct combination of primary and secondary diagnoses in the data, which is comparable with the simulation of Table 1 (0.05 sample/combination). In such sparse data, the conventional calculation of mutual information is easy to over-estimate while the proposed method provides relatively robust estimations. Therefore, we can reasonably consider that the estimation of the proposed method will be closer to the true mutual information.

**Figure 5:**
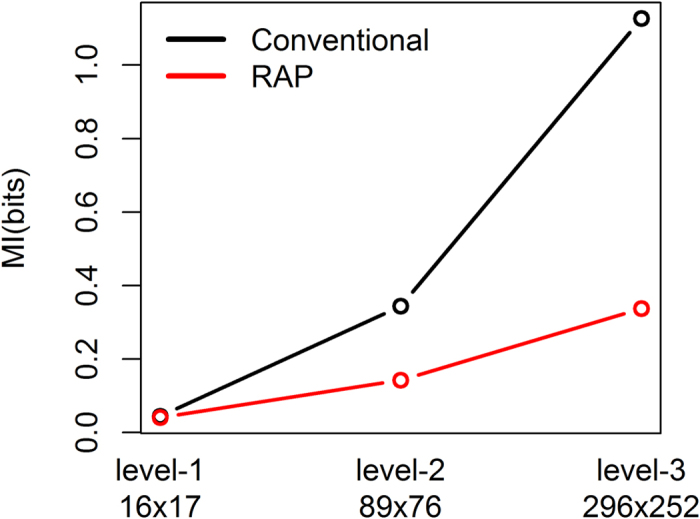
Mutual information between the primary and secondary diagnoses of the ICU patients. Shown are the mutual information values calculated from 296 × 252 3-digit ICD9 codes (level-3 categories) as well as 17 × 16 level-1 and 89 × 76 level-2 categories, by the proposed method with *p*-value threshold 0.5 (red) and the conventional method (black).

In addition, we evaluated the mutual information calculations in the hierarchy of the ICD9 codes. According to the similarity of disease signs, symptoms, and causes, the 3-digit ICD9 codes are further classified into fewer super categories. For example, ICD9 codes corresponding to ‘Cholera’, ‘Shigellosis’, ‘Amebiasis’, and other 6 diseases (level-3 category) are grouped into level-2 ‘Intestinal Infectious Diseases’ category, which is further grouped into level-1 ‘Infectious and Parasitic Diseases’ category with other 15 level-2 super categories. The hierarchy of the ICD9 code system has 17 level-1 super categories except supplementary codes. Among them level-1 ‘Infectious and Parasitic Diseases’ category has 138 level-3 categories, each of which corresponds to a 3-digit ICD9 code. In the ICU data here, 296 categories of the primary diagnoses are grouped into 89 level-2 categories, and further into 16 level-1 categories. Similarly, the secondary diagnosis has 252 level-3, 76 level-2, and 17 level-1 categories. Figure 5 shows the estimated mutual information values between the primary and secondary diagnoses with level-1, 2, and 3 categories by the proposed and conventional methods. The proposed method has relatively less changes across the different levels of categories while the conventional calculation has dramatic changes. Since the level-1 and 2 super categories partially reflect the hidden data structure of the level-3 fine categories by grouping diseases according to their similarity, the mutual information values with different categories are not expected to have huge difference. Therefore, this analysis also supports the robustness of the proposed method.

The mutual information was also estimated with various p-value thresholds (Supplementary Figure S2). The estimated mutual information was closer to the result of the conventional method with a higher p-value, which is a similar pattern with the cases of the simulation studies. This result is expected because the sample space becomes easier to be partitioned with higher p-values. Also as shown in Figs 2-4, the MI calculated by the proposed method depends on the p-value thresholds. For practical applications, we can determine the proper threshold using the results of intensive simulation studies such as Table 1. Even though, it is still important to study a parameter-free way of estimating the threshold.

The structure learned by the proposed method brings interesting insights for the ICU diagnosis data. First, the learned structure identifies sparse regions with rare samples. A large block with 234 primary and 157 secondary codes has only 44 samples. The combinations of this block have only one or zero sample. The density is 0.001 sample/combination, which is much lower than the average density (=0.066). In addition, 12 blocks have lower densities than the average density. The rare samples of these blocks have little contribution to the analysis of the data, and they might be ignorable in the data analysis. Second, some diseases tend to be partitioned together in the secondary diagnoses. For example, ‘Cardiac Dysrhythmias’ and ‘Heart Failure’, which are closely related and have the same level-2 category, are often partitioned into one block as the secondary diagnoses for the primary diagnoses of ‘Acute Bronchitis and Bronchiolitis’, ‘Acute and Subacute Endocarditis’, ‘Chronic Bronchitis’, and so on. More interestingly, ‘Heart Failure’ is often grouped together with ‘Other Diseases of Lung’ while cardiovascular and respiratory diseases are not close at all in the disease classification. It can be also observed for ‘Acute Kidney Failure’ and ‘Symptoms Involving Cardiovascular System’. Even with different disease signs and symptoms, the diseases grouped together for the secondary diagnoses might have a common underlying mechanism according to the primary diagnoses, which might be worth to be further studied.

## Conclusion

In this paper, we propose a method for the calculation of mutual information between discrete variables with many categories. By adaptively partitioning a sample space into subregions with uniform distributions, the proposed method robustly provides estimation for mutual information. Through the simulation studies for various types of data, the proposed method was shown to have superior accuracy and robustness to the sample size and the number of categories, compared with the conventional calculation of mutual information. The average error was reduced by 45 folds (12.20 to 0.27), and the correlation coefficient with true values was improved by 99 folds (0.01 to 0.99). In a case study to investigate the relation between the primary and secondary diagnoses of ICU patients, the proposed method provided reasonable mutual information without over-estimating the dependency between two diagnoses. The overall result demonstrates the benefits of the proposed method. In the future, we will further investigate the theoretical aspects of the method to guess the optimal p-value thresholds that can yield nearly perfect mutual information, which will be very useful for practical cases. We will also study a parameter-free way of estimating the p-value threshold. Another important issue is the efficient implementation of the method so as to overcome the high time complexity for the number of variable categories. We believe that the proposed method will be useful in the analysis of various biomedical data as well as data in the other areas of natural sciences, engineering, business, and social sciences.

## Additional Information

**How to cite this article**: Seok, J. and Kang, Y.S. Mutual Information between Discrete Variables with Many Categories using Recursive Adaptive Partitioning. *Sci. Rep.* **5**, 10981; doi: 10.1038/srep10981 (2015).

## Supplementary Material

Supplementary Information

## Figures and Tables

**Table 1:** Summary of the simulation tests.

	**(1)**	**(2)**	**(3)**	**(4)**	**(5)**	**(6)**	**Err.**	**Cor.**
True MI	0.09	0.61	0.14	0.53	0.16	0.43	N/A	N/A
Conventional	2.81	2.70	2.12	2.17	2.63	2.84	12.20	0.01
Random Partition	0.00	0.00	0.00	0.01	0.00	0.00	0.99	0.54
RAP, pv=0.1	0.02	0.31	0.10	0.24	0.09	0.12	0.55	0.91
RAP, pv=0.3	0.06	0.42	0.14	0.35	0.14	0.25	0.27	0.98
RAP, pv=0.5	0.14	0.52	0.21	0.54	0.22	0.43	0.27	0.99
RAP, pv=0.7	0.42	0.82	0.50	0.98	0.48	0.88	1.76	0.92
RAP, pv=0.9	2.02	2.01	1.62	1.91	1.97	2.52	8.98	0.35

For the simulation setting (1) to (6), shown are the mutual information values estimated from 500 samples when each variable has 100 categories. Shown are the averages of ratios of the errors to the true values (Err) as well as the correlation coefficients between the true and estimated values (Cor) across the six simulations.
