# Supplementary material for: Mutual Information between Discrete Variables with Many Categories using Recursive Adaptive Partitioning
Source: Sci Rep. 2015 Jun 5;5:10981. doi: 10.1038/srep10981 (PMC4456943; doi:10.1038/srep10981)
Supplement: Supplementary Information [file srep10981-s1.pdf]

Supplementary Information

Mutual Information between Discrete Variables with Many Categories using Recursive Adaptive Partitioning

Junhee Seok[1], Yeong Seon Kang[2*]

[1]School of Electrical Engineering, Korea University, Seoul, South Korea.
[2]Department of Business Administration, University of Seoul, Seoul, South Korea.

[*]Corresponding authors

Supplementary Methods

Simulation Settings

The proposed calculation of mutual information was evaluated through simulation studies. In each simulation, we considered a certain relation between two discrete variables where the true mutual information can be calculated. For two categorical variables X and Y whose relation was predefined, the mutual information was estimated from random samples. Six different relations were used for the simulation studies with various sample sizes (50, 100, 200, 500, 1,000, and 2,000), and numbers of categories per variable (2, 5, 10, 20, 50, and 100). Each simulation was repeated 100 times. The proposed methods with different p-value thresholds were compared with the true mutual information as well as the results of conventional calculation. All simulations are for the variables of which categorical values cannot be ordered.

Six simulation settings are listed here.

(1) Step structure with low mutual information: Considering X and Y with two super categories for each, i.e. $X \in \{w_1,w_2\}$ and $Y \in \{v_1,v_2\}$, their relation is given by 2 x 2 joint probabilities. We consider that $p(w_1,v_1)=0.4$, $p(w_1,v_2)=0.1$, $p(w_2,v_1)=0.2$, and $p(w_2,v_2)=0.3$. Here, we assume that $n/2$ fine categories per each super category are actually observed instead of the super categories. Let $x_1, \ldots, x_{n/2}$ be observed for w_1, and $x_{n/2+1}, \ldots, x_n$ be for w_2. Similarly, $y_1, \ldots, y_n$ are assumed to be observed for the super categories of Y. The combinations of the fine categories are assumed to be uniformly distributed within the corresponding combination of the super categories. For example, $(n/2)^2$ combinations of $\{x_1, x_2, \ldots, x_{n/2}\}\times\{y_1, y_2, \ldots, y_{n/2}\}$are uniformly distributed in $w_1\times v_1$ of which probability is 0.4. For the simulation, the mutual information of X and Y is estimated from randomly generated data with n fine categories per variable, and compared with the true mutual information, 0.09 bits.

(2) Step structure with high mutual information: This setting is similar with (1), but the joint probability of the super categories are given as $p(w_1,v_1)=0.7$, $p(w_1,v_2)=0$, $p(w_2,v_1)=0$, and

$p(w_2,v_2)$=0.3. Here, the true mutual information is 0.61 bits.

(3) Gaussian structure with low mutual information: The joint population of X and Y is defined by a bivariate joint Gaussian distribution, of which marginal distributions are standard and the covariance (σ) is 0.49. First, continuous random samples are generated from the joint distribution. The observed range of the continuous samples is uniformly discretized as n categories for each variable. Each sample falls into one of n^2 combinations of discretized X and Y, and has corresponding categorical values for X and Y. From the data with n categories per variable, the mutual information is estimated. Different from continuous variables, here the Gaussian structure is hardly observed because the discretized categories are observed without orders. When the marginal variance is 1, the theoretical mutual information of a joint Gaussian distribution is given as $\log(1/(1-\sigma^2))$, which is 0.14 bits in this case.

(4) Gaussian structure with high mutual information: This setting is similar with (3), but the covariance of X and Y is given as 0.81. The true mutual information is 0.53 bits.

(5) Random structure with low mutual information: A random relation between X and Y can be constructed by randomly generated joint probability masses. The probabilities of n categories of X are generated from an exponential distribution with λ=1. Let $\mathbf{p_X}$ denote the vector of these n probability masses. Similarly, the marginal probability mass vector of Y, $\mathbf{p_Y}$, is randomly generated from the same exponential distribution. We obtain an n x n matrix of the joint probability distribution, $\mathbf{P_1} = \mathbf{p_X}\mathbf{p_Y}^T$. $\mathbf{P_1}$ is the joint probability masses of a randomly structured but independent relation. Independently, we obtain $\mathbf{P_2}$, another n x n probability matrix, by randomly generating n^2 joint probability masses from an exponential distribution (λ=1). $\mathbf{P_2}$ represents a randomly structured and dependent relation. To ensure random structure and dependency, $(\mathbf{P_1}+\mathbf{P_2})/2$ is used for the final joint probability distribution. Samples are randomly generated by the final joint probabilities, from which the mutual information is estimated. Although the theoretical mutual information is hard to be obtained in this case, it can be empirically estimated with many samples (one million in this work). The true mutual information is expected to be different as the number of categories.

(6) Random structure with high mutual information: This setting is similar with (5), but only $\mathbf{P_2}$ is used for the final probabilities. In this case, *X* and *Y* are more dependent to each other than (5). Consequently, this setting simulates a random structure with higher mutual information than (5).

Time Complexity

The number of samples in the input data (*n*) is often used as the input data size for the calculation of time complexity. In the proposed algorithm, the number of categories of input discrete variables (*d*) can be considered as another input data size of interest. Here, we calculate the time complexity for each input data size, *n* and *d*. Consider the worst case that the whole sample space is partitioned into the finest combinations of categories of two variables. At every partitioning the marginal population should be calculated, which is simply counting samples. Since the number of partitions only depends on the number of categories in the worst case, the overall complexity as the number of samples *n* is O(*n*). To derive the time complexity for the number of variable categories *d*, assume that two variables can have *d* categorical values. At every partitioning, categorical values should be sorted, of which complexity is $k\log k$ when there are *k* categories in a subregion. In the worst case, *k* categorical values are partitioned into two subregions with 1 and *k*-1 categories, respectively. Therefore, the overall complexity for the number of categories *d* is $\mathrm{O}\left(\sum_{k=2}^{d-1} k \log k\right)$.

Supplementary Table S1. Available data sets of discrete variables with many categories.

Data set	Description	Variable	Number of Categories	Sample Size
MIMIC2[1]	Clinical records in intensive care	Diagnosis of admission	~300	~5,000
Medicare[2]	Medicare records in the US (1990-1993)	Inpatient claim	~1,000	~32M
STRIDE[3]	Electronic health records of Stanford hospitals (2005-2010)	Inpatient claim	~1,000	~2.7M
		Medical operation	~1,000	~3.1M
T-cell repertoire[4]	Short sequencing reads of T-cell receptors mapped to specific V, D and J segments	V segment	~80	~1M
		D segment	~30	
		J segment	~6	
PheWAS[5]	Phenome-wide association study	Disease group	~1,500	~13,000
		Genotype of *n* SNPs	3^n	

[1]Scott, D. J. *et al.* Accessing the public MIMIC-II intensive care relational database for clinical research. *BMC Med Inform Decis Mak* **13**, 9 (2013).
[2]Hidalgo C.A. *et al.* A Dynamic Network Approach for the Study of Human Phenotypes. *PLoS Computational Biology*, 5(4):e1000353 (2009).
[3]https://med.stanford.edu/clinicalinformatics.html
[4]Georgiou, G. *et al.* The promise and challenge of high-throughput sequencing of the antibody repertoire. *Nat Biotechnol* **32**, 158-168 (2014).
[5]Denny, J. C. *et al.* PheWAS: demonstrating the feasibility of a phenome-wide scan to discover gene-disease associations. *Bioinformatics* **26**, 1205-1210 (2010).

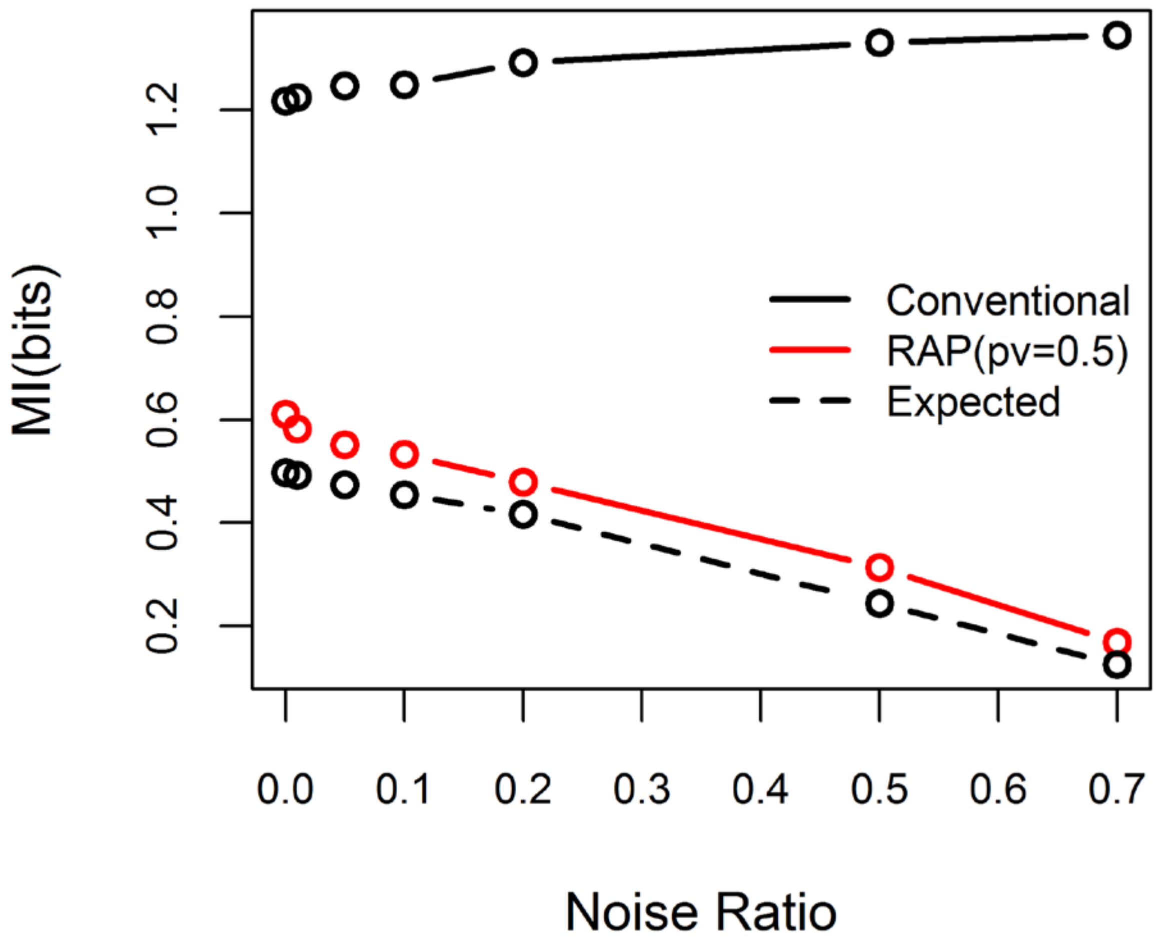

Supplementary Figure S1. Noise effects on mutual information. Uniformly distributed noise was added to samples generated by the simulation setting (4) in the main text. The x-axis is the ratio of noise samples among the whole samples. Shown are the expected mutual information (black dashed) as well as estimations by the proposed method (red solid) and the conventional method (black solid).

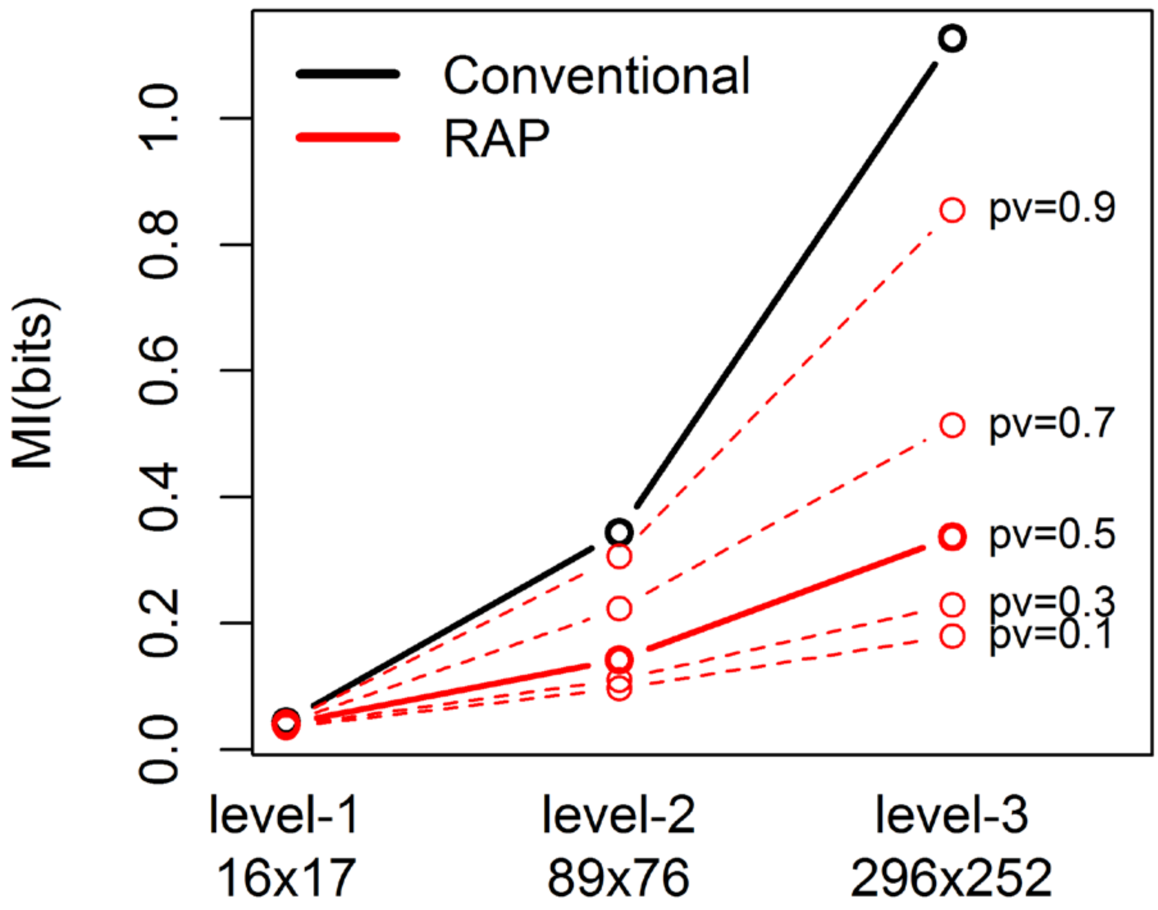

Supplementary Figure S2. Mutual information of the ICU data with various p-value thresholds. Shown are the mutual information values calculated from 296 x 252 3-digit ICD9 codes (level-3 categories) as well as 17x16 level-1 and 89 x 76 level-2 categories, by the proposed method with *p*-value threshold 0.5 (red) and the conventional method (black). The results of the proposed method with various p-value thresholds are also shown (red dashed).
